# Antennal-Expressed Ammonium Transporters in the Malaria Vector Mosquito *Anopheles gambiae*

**DOI:** 10.1371/journal.pone.0111858

**Published:** 2014-10-31

**Authors:** R. Jason Pitts, Stephen L. Derryberry, Fadi E. Pulous, Laurence J. Zwiebel

**Affiliations:** 1 Department of Biological Sciences, Vanderbilt University, Nashville, Tennessee, United States of America; 2 Vanderbilt Institute for Global Health, Nashville, Tennessee, United States of America; 3 Department of Pharmacology, Vanderbilt Brain Institute, Program in Developmental Biology and Institute of Chemical Biology, Vanderbilt University Medical Center, Nashville, Tennessee, United States of America; Centro de Pesquisas René Rachou, Brazil

## Abstract

The principal Afrotropical malaria vector mosquito, *Anopheles gambiae* remains a significant threat to human health. In this anthropophagic species, females detect and respond to a range of human-derived volatile kairomones such as ammonia, lactic acid, and other carboxylic acids in their quest for blood meals. While the molecular underpinnings of mosquito olfaction and host seeking are becoming better understood, many questions remain unanswered. In this study, we have identified and characterized two candidate ammonium transporter genes, *AgAmt* and *AgRh50* that are expressed in the mosquito antenna and may contribute to physiological and behavioral responses to ammonia, which is an important host kairomone for vector mosquitoes. *AgAmt* transcripts are highly enhanced in female antennae while a splice variant of *AgRh50* appears to be antennal-specific. Functional expression of AgAmt in *Xenopus laevis* oocytes facilitates inward currents in response to both ammonium and methylammonium, while AgRh50 is able to partially complement a yeast ammonium transporter mutant strain, validating their conserved roles as ammonium transporters. We present evidence to suggest that both *AgAmt* and *AgRh50* are *in vivo* ammonium transporters that are important for ammonia sensitivity in *An. gambiae* antennae, either by clearing ammonia from the sensillar lymph or by facilitating sensory neuron responses to environmental exposure. Accordingly, *AgAmt* and *AgRh50* represent new and potentially important targets for the development of novel vector control strategies.

## Introduction

Gaseous ammonia and its protonated ionic form, ammonium, (collectively referred to as ammonium) are important molecules for life on earth. For many organisms, ammonium is a critical precursor for a wide range of biologically active macromolecules. For example, plants cannot utilize atmospheric nitrogen gas and must fix nitrogen from the soil via symbiotic bacteria and fungi [1], [2]. While some organisms do not uptake ammonium directly, they import other nitrogen sources and convert them into ammonium for use in biosynthetic pathways [3]. Paradoxically, while ammonium uptake is crucial for biological systems, it is also produced as a waste product of nucleic acid and amino acid metabolism and is toxic at high concentrations [4]. It is therefore not surprising that regulation of ammonium levels at cellular, organ, tissue, and organismal levels is paramount to viability of virtually all forms of life. Cells have devised a number of mechanisms to deal with excess ammonia, including conjugation of amines to larger non-toxic compounds and secretion of ammonium in various forms [4]–[6]. The latter process requires the action of transmembrane proteins that increase the permeability of ammonium across cell membranes, and are accordingly classified as ammonium transporters [6], [7]. Diverse mechanisms have evolved for the transport of ammonium and ammonium derivatives in cells. These include the ammonium transporters (Amt) in bacteria and plants, the methylammonium/ammonium permeases (MEPs) in yeast, and the Rhesus (Rh) proteins in mammals [6], [8], [9]. In microbes, a role for ammonium transporters in sensing environmental levels of ammonia has been described [10]–[13].

Canonically, ammonium transporter proteins have been thought to facilitate the movement of ammonium ions across cell membranes [6], [9]. After ammonium is accumulated inside, it is used for metabolic purposes such as in the synthesis of biological macromolecules. However, there has been support from various studies involving fungi and bacteria that indicate that ammonium transporters may additionally function as ammonium sensors [14]. In *Saccharomyces cerevisiae*, *mep2* null mutants do not exhibit wild-type levels of pseudohyphal growth upon nitrogen starvation that is thought to occur as the cell is searching for a source of nutrients [10]. Researchers later concluded that MEP2 was necessary but not sufficient for the production of these filamentous growths under nitrogen-limiting conditions [10]. In a similar manner, AmtB from *Escherichia coli* is regulated by GlnK, a P_II_ class signal transduction protein [15]. When ammonium is sparse, GlnK is in its fully-uridylylated state and is not membrane associated; however, in conditions when ammonium concentrations are high, GlnK is deuridylylated and associates tightly with AmtB causing ammonium flux to stop [12]. An intriguing possibility is that as a result of their ability to facilitate ammonium flux across cell membranes eukaryotic ammonium transporters also act as either direct, or indirect chemosensors. While ammonium transporters have been described in numerous species, little is known about the specifics of their expression and functionality in insects.

In *An. gambiae* and other insects, three families of ligand-gated receptors are known to participate in various aspects of chemosensation: the odorant receptors (AgOrs), the variant ionotropic glutamate receptors (AgIrs), and the gustatory receptors (AgGrs) [16]–[18]. The Ors are primarily expressed in the olfactory receptor neurons (ORNs) that are housed within sensilla extending from the surfaces of the adult head appendage: antennae, maxillary palps, and proboscises [17], [19], [20]. They function primarily as ionotropic receptors that mediate sensitivities to volatile environmental odors that are carried by air currents in the near, or even distant, vicinity of the mosquito [21]–[24]. The well-conserved coreceptor, Orco, plays a vital role in mediating Or localization and function in a broad array of insect taxa [25]. The Grs are sensors of soluble compounds that include sugars, bitter tastants, and contact pheromones and are broadly expressed in the proboscises and leg tarsi of insects [26]–[31]. In adult *An. gambiae*, the major components of human sweat, such as ammonia, lactic acid, and carboxylic acids activate ORNs found in trichoid and basiconic (grooved peg) sensilla [32], the latter of which lack AgOrs [19]. While AgOrs are likely to account for a significant component of the molecular basis of trichoid sensitivities, the broad gaps in AgOr-based odor coding [33], [34] suggest an alternative molecular foundation for grooved peg sensitivities. Studies in *Drosophila melanogaster* [35]–[38] and *Schistocerca gregaria* [39] indicate that Irs are expressed in grooved peg sensilla and mediate their responses to chemical odors. By inference, the AgIRs are the most likely candidates to mediate grooved peg physiological responses in *An. gambiae* [18], [40]. Considering the importance of ammonia and amine compounds as human-derived kairomones affecting *An. gambiae* host-seeking behavior, identifying sensory receptors as well as salient ancillary proteins that are involved in the perception of these compounds would both improve our understanding of *An. gambiae* chemosensory mechanisms and provide additional targets for intervention strategies that could lower the incidence of human malaria transmission.

Even with the current advances in medical and other technologies, malaria remains a significant threat to global health [41]. *Anopheles gambiae*, the sub-Saharan human malaria vector mosquito, has been responsible for millions of deaths worldwide as a consequence of its anautogenous lifestyle. Human host odors, including those found in analyses of human sweat (ammonium, lactic acid, various carboxylic acids), are able to elicit electrophysiological responses in the adult female mosquito antennae [32], [40], [42]–[44]. Despite understanding which sensilla are responsive to ammonium, the molecular basis for *An. gambiae*'s sensitivity to ammonia remains unknown [32].

We have previously utilized next generation RNA sequencing to examine the transcriptome profile from chemosensory tissues of *An. gambiae* [45], [46]. In addition to numerous other transcripts with similar expression profiles, we have identified 2 candidate ammonium transporters, *AgAmt* and *AgRh50*, whose transcripts are highly abundant in the antennae of *An. gambiae*. We postulate that enhanced antennal expression indicates that *AgAmt* and *AgRh50* play important roles in the peripheral chemosensory signal transduction pathways that detect environmental ammonia in *An. gambiae*. *AgAmt* and *AgRh50* transcripts were also found in whole bodies of both females and males, consistent with their roles in ammonia clearance in Malpighian tubules and other body tissues. We have employed heterologous expression systems to confirm that *AgAmt* and *AgRh50* are indeed functional ammonium transporters. The successful functional characterization of *An. gambiae* ammonium transporters strengthens our basic understanding of chemosensory processes in mosquitoes, and accordingly, may have profound implications for global health.

## Materials and Methods

### Ethics Statement

This study adhered to the Guide for the Care and Use of Laboratory Animals of the National Institutes of Health. The use of *Xenopus laevis* for oocyte harvesting was approved by the Vanderbilt University Institutional Animal Care and Use Committee (Protocol M/10/174). Surgeries were performed under Tricaine methanesulfonate (MS-222) and cold anesthesia using aseptic technique. Every effort was made to minimize pain and suffering.

### Gene Identification and Phylogeny

Ammonium transporter sequences (AGAPs 003989 and 002011) in the *An. gambiae* genome annotation v3.7 were queried in tBLASTn and BLASTp searches using default parameters (http://www.vectorbase.org) to identify other homologous sequences, but no other significantly similar hits were found. Conceptual exons and intron were identified using the Softberry HMM gene search software (http://www.softberry.com). Protein sequences were aligned with Clustal X software [57]. Neighbor-Joining trees [58] were constructed from peptide alignments with 100 bootstrap replicates. Transmembrane plots were generated using the Protter protein display software [59].

### Reverse Transcription/Polymerase Chain Reaction

*AgAmt* and *AgRh50* transcript levels were determined by means of quantitative RT-PCR. Each sample was comprised of 10 heads, 50 antennae, 50 palps or 10 carcasses that were hand-dissected from batches of control and experimental 5–7 d.o. *An. gambiae* adult females. RNA was extracted using the trizol method as described [45] and cDNA synthesis were performed using the Transcriptor First Strand cDNA Synthesis Kit (Roche, Inc.). All primers in the assay were designed to span predicted introns in order to distinguish well between genomic DNA and cDNA templates. *An. gambiae* ribosomal protein lysosomal aspartic protease (AGAP003277; *AgLap*), which is constitutively expressed at high levels in all tissues, was chosen as control gene to measure the levels of *AgAmt* and *AgRh50* mRNA *in vivo*. Primer sequences are as follows: *AgLap* forward 5′-CCAACTATCTCGATGCTCAAT-3′; *AgLap* reverse 5′-ATTCTTCTCGAACGAGGACG-3′ (product size: 188 bp); *AgAmt* forward 5′-GGAAGTTTCAGCATCATCTATT-3′ and reverse 5′-CCGACGGCTAGCACACCCCA-3′ (product size: 266 bp); *AgRh50a* forward 5′-GCGTCGAAACATACGGCACC-3′ and *AgRh50a* reverse 5′-GAGGGTGATTTGAGTATCAATCCGG-3′ (product size: 233 bp); *AgRh50b* forward 5′-GGCGGGCTGATAACCGGAGTGA-3′ and *AgRh50b* reverse 5′-GAGGGTGATTTGAGTATCAATCCTT-3′ (product size: 213 bp). PCR amplicons were cloned and sequenced to confirm their identities. qRT-PCR was carried out using an Applied Biosystems 7300 Real-time PCR system and SYBR green as fluorescent dye. Three experimental repetitions were analyzed for each biological sample and the data processed using System 7300 Sequence Detection Software (version 1.3.1). Primer efficiency was determined using a standard curve for all the primers used. In the amplification of *AgAmt* and *Rps7*, 8 µl and 2 µl cDNA, respectively, from each group were used as templates. In each trial, *AgAmt* or *AgRh50* cDNA levels were quantified relative to *AgLap* levels using the method of Pfaffl [60].

### RNA Sequencing

*Illumina* RNA-seq read files from a previous study [45] were derived from *An. gambiae* tissues and are freely available at the National Center for Biotechnology Information Sequence Read Archive (http://trace.ncbi.nlm.nih.gov/Traces/sra/; SRA study accession: SRP028873). Total weighted reads and transcript lengths for *AgAmt*, *AgRh50a*, and *AgRh50b* were used to calculate normalized transcript abundance levels in units **R**eads **P**er **K**ilobase per **M**illion reads mapped (RPKM) [61].

### cDNA Vector Construction and cRNA synthesis

The full-length coding sequences of *AgAmt* and *AgRh50* were amplified by polymerase chain reaction from a cDNA library derived from *An. gambiae* antennae using primers specific for their coding sequences: *AgAmt* forward 5′-CACCATGGCCAACGGCACAACGATGG-3′; *AgAmt* reverse 5′-TTATCAATCCACGCAAAGTT-3′; *AgRh50a/b* forward 5′-CACCATGCACACACCAGGATCCTC-3′ *AgRh50a/b* reverse 5′-CTAGTTGGATGATTCGTTGGT3′. Once amplified, *AgAmt*, *AgRh50a* and *AgRh50b* were cloned into pSP64T-Oligo expression plasmid using the Gateway System^R^ (Invitrogen). cRNA for was synthesized from linearized plasmid (*Xba*I digest) using the mMessage mMachine^R^ SP6 RNA polymerase kit (Life Technologies, Inc.).

### Xenopus laevis Oocyte Retrieval, Microinjection, and Incubation

Expression and functional characterization of *AgAmt* was carried out essentially as described for *AgOR* proteins [17], [34], [62]. *X. laevis* ovarian lobes were extracted from fully mature females via laparotomy. An incision less than 1 cm was made ventrally, lateral of the midline, in the organism's skin, and then the ovarian wall. The ovarian lobes were then extracted and placed in a washing buffer (96 mM NaCl, 2 mM KCl, 5 mM MgCl_2_, and 5 mM Hepes, pH 7.6) supplemented with gentamycin. The ovarian lobes were gently separated via forceps and subsequently treated with 2 mg/mL collagenase S-1 in washing buffer for 45 minutes to an hour at room temperature while on a laboratory shaker. Oocytes were then washed twice with washing buffer and incubated in ND96 buffer (96 mM NaCl, 2 mM KCl, 1 mM MgCl_2_, 1 mM CaCl_2_, and 5 mM Hepes, pH 7.5) supplemented with 5% dialyzed, heat-inactivated horse serum, 50 µg/mL tetracycline, 100 µg/mL streptomycin, and 550 µg/mL sodium pyruvate at 18°C until microinjection.

Each oocyte was selected for injection based on health (appearance) and developmental stage (stage V or VI). Each oocyte was injected with approximately 30 ng *AgAmt* cRNA. After injection, oocytes were incubated separately at 18°C in supplemented ND96 buffer. Oocytes were allowed to incubate for 2 to 5 days until assayed by two-electrode voltage clamp electrophysiology.

### Solutions

Salts of the highest commercial grade available were obtained from Fisher Scientific, Alfa Aesar, or Sigma Aldrich. 1M chloride salts, originally diluted in ND96, were diluted further in ND96 to reach the tested concentrations. During the TEVC and voltage-step assay, ND96 was continuously perfused over each oocyte. Different stimuli were introduced using the ValveLink 8.2 stimulus controller (AutoMate Scientific).

It is known that ammonium (NH_4_^+^) will induce endogenous currents in *X. laevis* oocytes when the concentration of the species is equal to or greater than 1 mM [47], [48]. Most solutions used in these experiments did not exceed a concentration of 200 µM ammonium to monitor the effects that ammonium has on *AgAmt* and not endogenous proteins found in the oocyte; however, to observe whether the contribution of *AgAmt* was greater than the endogenous currents elicited by ammonium concentrations greater than 1 mM, the magnitude of the currents resulting in water-injected oocytes were compared to those resulting in *AgAmt*-injected oocytes.

### Two-Electrode Voltage Clamp Electrophysiology

Whole-cell currents were recorded from injected oocytes using the OC-725C feedback amplifier (Warner Instruments). The amplifier was connected to a computer interface through the Axon Digidata 1440A Digitizer; data acquisition and analysis were carried out using pCLAMP 10 software (Axon Instruments). The current-injecting and voltage-sensing electrodes were made of Ag/AgCl wires housed in pulled borosilicate glass capillaries each containing 0.2 µM filtered 3M KCl. A salt bridge (3M KCl and 4% w/v agarose) connected the bath solution and the sense electrode. Each oocyte was clamped at a holding potential of −80 mV, excluding current-voltage analysis. Stimuli were delivered to oocytes until steady state currents developed. The resulting magnitude of the change in current before the stimulus and steady state was calculated using pCLAMP 10 software and was subsequently used for data analysis.

### Voltage-Step Assay

Current versus voltage plots were generated using the following protocol. Stimuli were continuously applied to an oocyte voltage-clamped at −100 mV. After approximately 30 seconds (the time required to reach steady state), a voltage-step protocol was executed in which the voltage increased by 10 mV with each run, starting at −100 mV and ending with +40 mV. Each run lasted 200 milliseconds in order to minimize the effect of rectifying currents. Current magnitudes were calculated by calculating the absolute difference of the currents measured for a stimulus at a given voltage (either ammonium chloride or methylammonium chloride) from the currents resulting from the perfusion of ND96 at those same voltages.

### *Yeast Transformation and Complementation*

*S. cerevisiae* yeast strains were obtained from the laboratory of Dr. Anna Maria Marini (Université Libre de Bruxelles). Strain 31019b is a triple mutant of the methyl ammonium permeases *mep1*, *mep2* and *mep3* (*mep1-3Δ*) and strain 23344c is the wild type background *ura3* deletion strain [63]. *An. gambiae* ammonium transporter coding sequences were cloned into yeast expression plasmid, pAG426GAL-ccdB (www.Addgene.org; plasmid 14155) using the LR Clonase system (Invitrogen) and verified by PCR, restriction digest analysis and Sanger sequencing. Yeast transformations were carried out using the polyethylene glycol and lithium chloride method as previously described [64]. Selection for transformed yeast was performed on solid medium without uracil, plus 0.17% yeast nitrogen base (YNB) lacking amino acids and ammonium sulfate (Difco #Y1251), supplemented with 2% galactose and 1 mM Arginine as the sole nitrogen source. Transformants were verified by PCR using gene specific primers as described above.

Yeast strains were cultured in liquid medium containing 1 mM Arginine as the sole nitrogen source until optical density at 600 nm (OD_600_) reached 0.6–0.8. Cells were pelleted by centrifugation, washed in sterile water, and resuspended in fresh medium to an initial OD_600_ of 0.05 in liquid culture or 10 ul droplets of 1000–2000 cells per spot on solid media. Growth at 30°C was then monitored every 24 hours in 1 mM ammonium sulfate or Arginine for each transformant and compared with controls; wild type or *mep1-3Δ* transformed with the empty pAG426GAL-ccdB plasmid. Growth assays were repeated 4 times for liquid and solid media. Mann-Whitney U tests were conducted as a non-parametric test of means comparing average OD_600_ at 4–7 days post-inoculation for liquid cultures of wild type and *An. gambiae* ammonium transporter transformants against *mep1-3Δ*. Colonies growing on solid media at 2–4 days post-inoculation were photographed with the aid of a dissecting microscope and digital camera. Individual colonies located within 10 ul circular spots were analyzed using ImageJ software [65] to determine average area in square millimeters.

## Results

### *An. gambiae* Ammonium Transporter Genes

**Figure 1:**
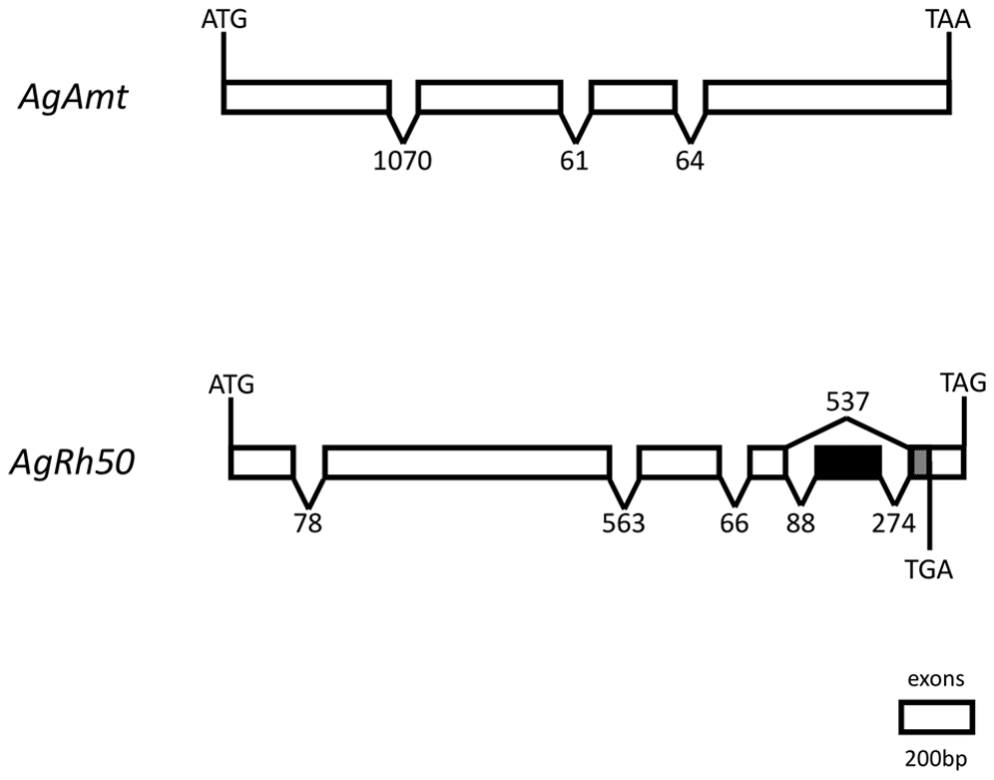
*An. gambiae* ammonium transporter gene structures. Schematic representation of the exon (boxes) and intron (lines) structure of *AgAmt* and *AgRh50* genomic loci. Alternative splicing at 3′ end of *AgRh50* indicated by white boxes (*AgRh50a*) and black box and shaded region of final exon (*AgRh50b*) and TAG and TGA stop codons, respectively. Scale bar is for exons only; introns lengths in base pairs are indicated above or below splice sites.

**Figure 2:**
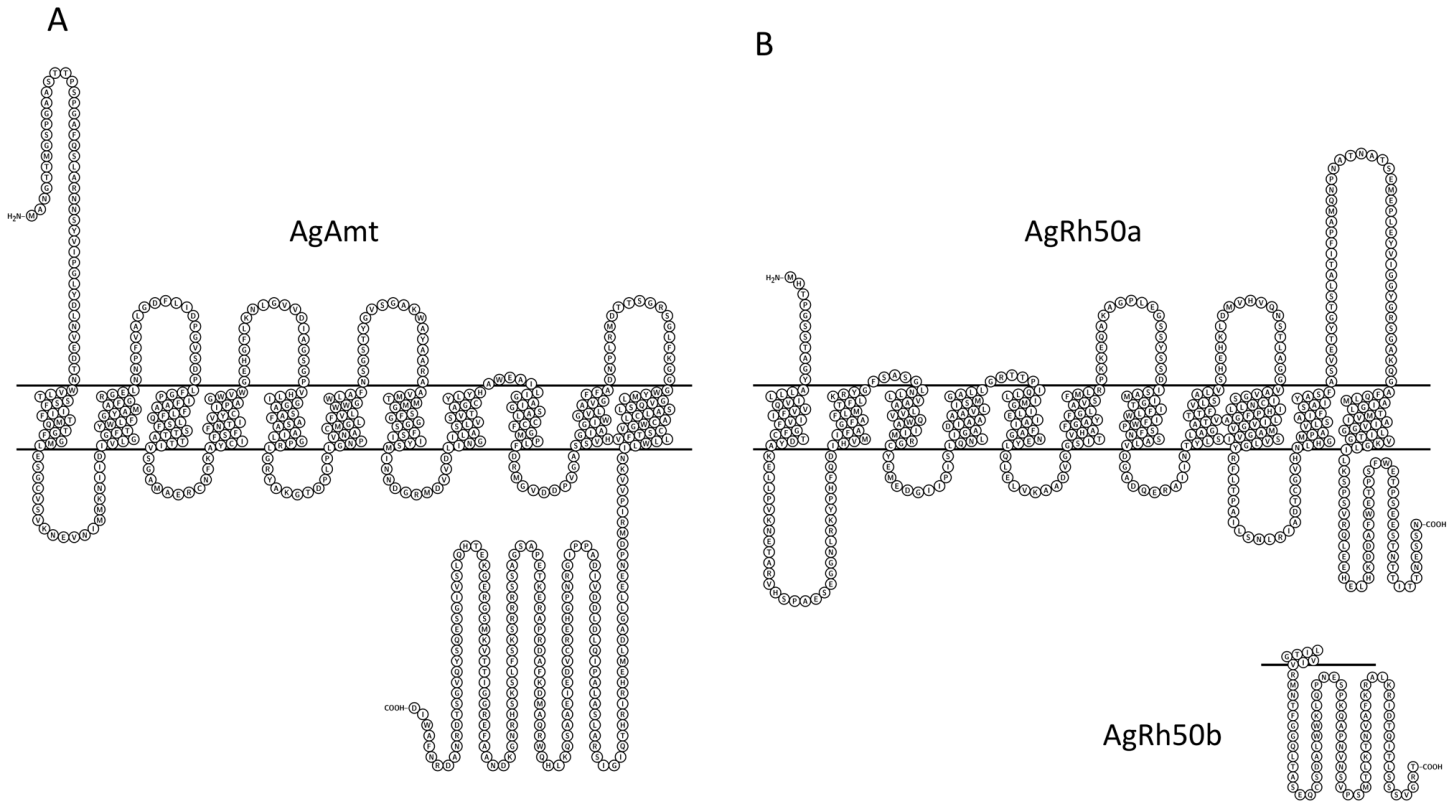
Amino acid plots of AgAmt (A) and AgRh50 (B) depicting the 11 transmembrane domains. AgRh50a and AgRh50b differ only in their C-terminal regions.

**Figure 3:**
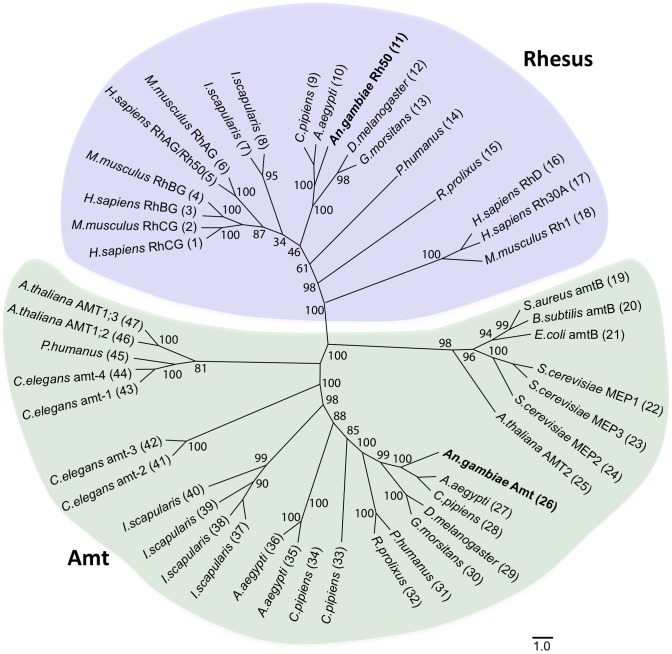
Phylogenetic tree of ammonium transporter families. Neighbor-joining tree comparing relationships among ammonium transporter proteins from prokaryotes and eukaryotes. Rhesus (blue shaded region) and Amt (green shaded region) proteins segregate into distinct families, with insects having representatives in both groups. Bootstrap support for branches is indicated. Scale bar is 1% corrected distance. Sequences and species names are found in Table S1.

The *An. gambiae* genome encodes two candidate ammonium transporter genes, AGAP003989 and AGAP002011, one representative from each of the Amt and Rhesus subfamilies, which we have named *AgAmt* and *AgRh50*, respectively (Figure 1). *AgAmt* appears to encode a single transcript (Figure 1) and a conceptual peptide of 591 amino acids (Figure 2A). *AgRh50* appears to encode 2 alternatively spliced transcripts, *AgRh50a* and *AgRh50b* (Figure 1), the latter containing a novel 175 bp exon near the 3′ end of the transcript that was not predicted in the *An. gambiae* genome annotation (Figure 1). The transcripts for *AgRh50a* and *AgRh50b* encode conceptual peptides of 470 and 497 amino acids, respectively (Figure 2B). In addition, both peptides are predicted to form 11 transmembrane helices with an extracellular N-terminus and intracellular C-terminus (Figure 2), similar to other known ammonium transporters [47]. AgAmt and AgRh50 are clearly distinguishable as members of the two major ammonium transporter subfamilies (Figure 3) and share significant homologies with proteins encoded in several other insect genomes that are also putative ammonium transporters (Figure 3, Table S1).

### AgAmt and AgRh50 Transcript Abundances

**Figure 4:**
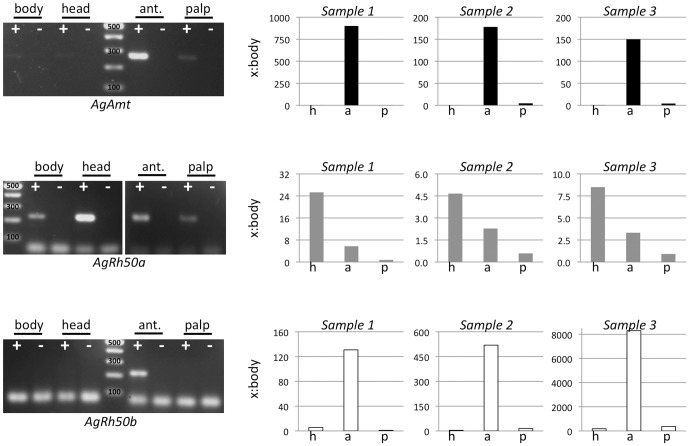
*An. gambiae* ammonium transporter expression in adult tissues. Left panels: RT-PCR amplification of *AgAmt*, *AgRh50a*, *AgRh50b* using cDNAs derived from adult female bodies, heads, antennae, and maxillary palps. +/− indicates presence or absence of reverse transcriptase in cDNA synthesis reactions. 100 bp marker shown for each gel image. Amplicons are of the expected sizes for each transcript: *AgAmt* (266 bp), *AgRh50a* (223 bp), *AgRh50b* (213 bp). Right panels: qRT-PCR amplification of *AgAmt*, *AgRh50a*, and *AgRh50b* relative to body (x:body) expression using *AgLap* as a normalizing transcript in each of 3 biological replicates (*Samples 1–3*). *AgAmt* (black bars) and *AgRh50b* (white bars) transcripts are dramatically enhanced in antennae (a), while *AgRh50a* (gray bars) transcript is enhanced in heads (h), minus antennae (a) and palps (p).

RNA sequencing and RT-PCR confirmed the presence of *AgAmt* and *AgRh50* transcripts in several adult tissues including antennae, maxillary palps, heads and whole bodies of *An. gambiae* (Table 1, Figure 4). Strikingly, transcripts for *AgAmt* and the *AgRh50b* splice variant were highly enriched in antennae compared with other tissues, while the *AgRh50a* transcript was much more evenly distributed in abundance (Table 1, Figure 4). Quantitative PCRs (qPCRs) confirmed the enhanced antennal specificity of *AgAmt* and *AgRh50b*, which were >100 fold overabundant in antennae relative to bodies (Figure 4). This analysis also revealed an enhancement (>4.5–24 fold) of *AgRh50a* transcripts in heads devoid of chemosensory appendages relative to bodies (Figure 4). While AgAmt and AgRh50b protein levels have yet to be determined in these tissues, enhanced levels of *AgAmt* and *AgRh50* transcripts in antennae are consistent with important roles for ammonium transporters in antennae function.

**Table 1:** Transcript abundance values for *An. gambiae* ammonium transporters in adult tissues (adapted from [45]).

	*AgLap*	*AgAmt*	*AgRh50a*	*AgRh50b*
body	296.9	0.6	5.0	3.5
palp	366.1	1.0	2.7	0.0
antenna	466.8	93.7	5.1	20.5

Ammonium transporter expression in female tissues. Values are given in RPKM (**R**eads **P**er **K**ilobase per **M**illion reads). *Ag*: *An. gambiae*; *Lap*: lysosomal aspartic protease; *Amt*: ammonium transporter; *Rh50*: Rhesus 50.

### TEVC Analysis of AgAmt-Injected Oocytes

**Figure 5:**
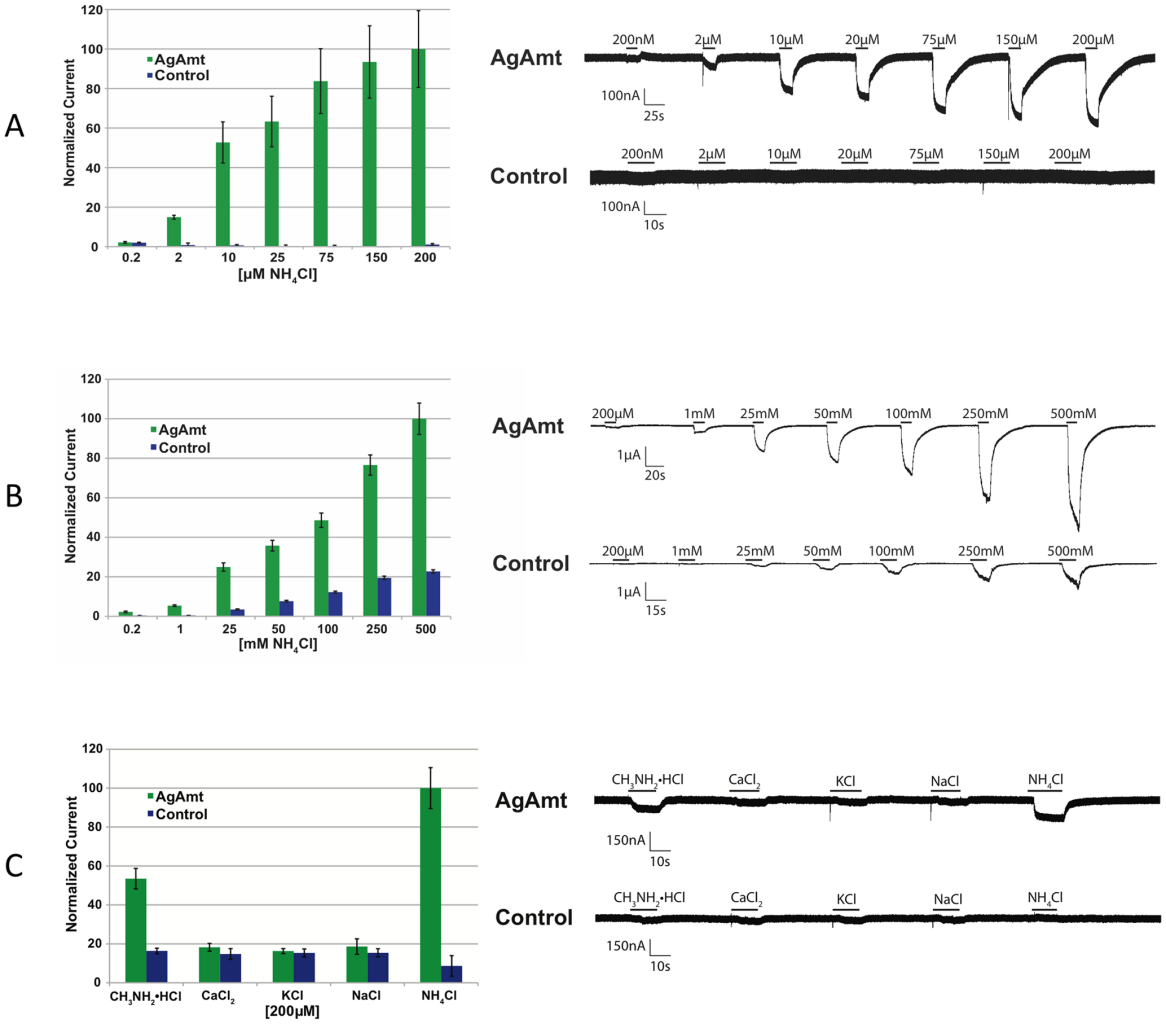
Function of AgAmt in *Xenopus* oocytes. Representative traces showing an *AgAmt* cRNA injected oocyte and water injected oocyte to concentrations of ammonium chloride. Black bars indicate stimulus length. Histogram showing normalized magnitude of inward current measured at steady state for *AgAmt* injected oocytes (green) and water injected oocytes (blue) to concentrations of ammonium chloride. Currents were normalized with respect to the average steady state current that resulted from *AgAmt* oocytes when stimulated with 200 µM ammonium chloride. *Higher Concentrations:* Representative traces showing an *AgAmt* cRNA injected oocyte and water injected oocyte to concentrations of ammonium chloride. Black bars indicate stimulus length. Histogram showing normalized magnitude of inward current measured at steady state for *AgAmt* injected oocytes (green) and water injected oocytes (blue) to concentrations of ammonium chloride. Currents were normalized with respect to the average steady state current that resulted from *AgAmt* oocytes when stimulated with 500 mM ammonium chloride. *Different Compounds:* Representative traces showing an *AgAmt* cRNA injected oocyte and water injected oocyte to different chloride salts at 200 µM. Black bars indicate stimulus length. Histogram showing normalized magnitude of inward current measured at steady state for *AgAmt* injected oocytes (green) and water injected oocytes (blue) to 200 µM chloride salt. Currents were normalized with respect to the average steady state current that resulted from *AgAmt* oocytes when stimulated with 200 µM ammonium chloride.

In order to determine whether *AgAmt* and *AgRh50* can form functional ammonium transporters, we used the *Xenopus laevis* heterologous expression system and two-electrode voltage clamp electrophysiology (TEVC). Oocytes injected with *AgAmt* cRNAs consistently evoked inward currents when voltage clamped at −80 mV in response to perfusions of ammonium chloride concentrations ranging from 200 nM to 200 µM (Figure 5A). As a negative control, buffer-injected oocytes were perfused with the same concentrations of ammonium chloride, yet no currents were observed (Figure 5A). A similar characterization of both splice forms of AgRh50 was attempted using the oocyte system; however, currents were not observed when AgRh50a or AgRh50b cRNA-injected oocytes were perfused with ammonium and methylammonium (data not shown). While we can reasonably conclude the observed inward currents from *AgAmt*-injected oocytes are likely due to the functional presence of *AgAmt* in the oocyte plasmid membrane, we cannot exclude the possibility that the observed currents are due to the interaction of the exogenously expressed transporter with endogenous proteins (Figure 5A). Previous studies have demonstrated that *X. laevis* oocytes produce endogenous inward currents in response to concentrations of ammonium at or above 1 mM [48], [49]. *AgAmt*-injected oocytes showed increasing responses to ammonium chloride at concentrations up to 500 mM that were always much larger in amplitude than endogenous currents recorded from water-injected controls (Figure 5B). These results suggest that the total whole cell current in *AgAmt*-injected oocytes is the summation of currents produced by the endogenously expressed ammonium transporters and the AgAmt transporter, which is responsible for the larger amplitudes compared to controls (Figure 5B).

Previously characterized ammonium transporters have the ability to transport a slightly larger derivative of ammonium, methylammonium [50]–[53]. To examine whether *AgAmt* is similarly able to transport this ion, 200 µM methylammonium chloride was perfused over voltage clamped *AgAmt*-injected oocytes. Inward currents in response to 200 µM methylammonium were approximately 55% of the magnitude of the inward currents recorded from the same oocytes perfused with 200 µM ammonium chloride (Figure 5C). In addition, other chloride salts were also perfused over *AgAmt*-injected oocytes to ascertain whether the observed inward currents were due to the presence of chloride anions. In these studies no inward currents were evoked in response to Na^+^, K^+^, or Ca^2+^ chloride salts at 200 µM (Figure 5C). As before, inward currents were not observed from water-injected controls to any of the tested chloride salts at 200 µM (Figure 5C). On the whole, these results are consistent with previous studies in that *AgAmt*-injected oocytes display inward currents in response to perfusions of ammonium and to a lesser magnitude with methylammonium, but fail to elicit inward currents with other ions [51], [54].

**Figure 6:**
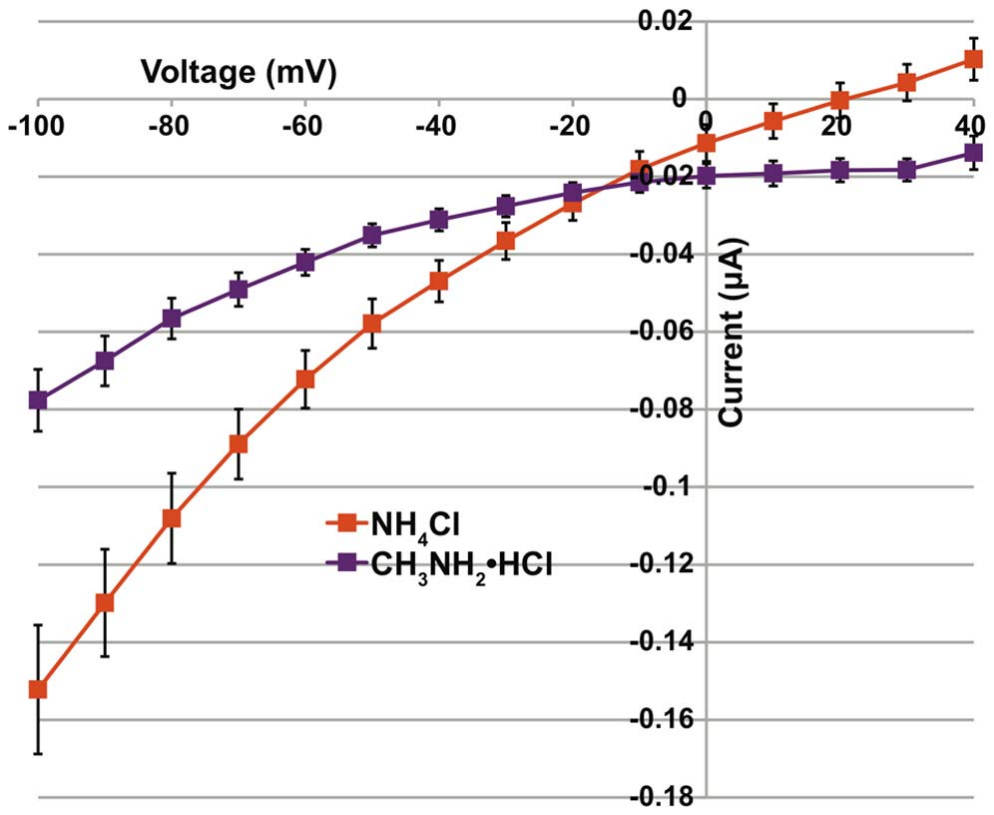
I–V Plot of whole cell conductances in oocytes expressing AgAmt. Current-voltage relationship for *AgAmt* cRNA injected oocytes to ammonium chloride (orange) and methylammonium chloride (purple). The x-axis shows voltage measured in millivolts and the y-axis shows current measured in microamps.

A current-voltage plot was generated by measuring ion-induced currents at several different membrane holding potentials in order to further examine the relationship between AgAmt-dependent ammonium and methylammonium conductances (Figure 6). The slope of current/voltage (I/V = conductance) in response to 200 µM ammonium chloride was much greater than the corresponding slope in response to 200 µM methylammonium chloride (Figure 6). This result clearly shows that ammonium elicits a larger whole-cell conductance than methylammonium at the same concentrations. In addition, the reversal potential of each ion, defined as the voltage at which the recorded current is zero, was approximately 20 mV for ammonium and >40 mV for methylammonium under these conditions.

### Yeast Ammonium Transporter Complementation

**Figure 7:**
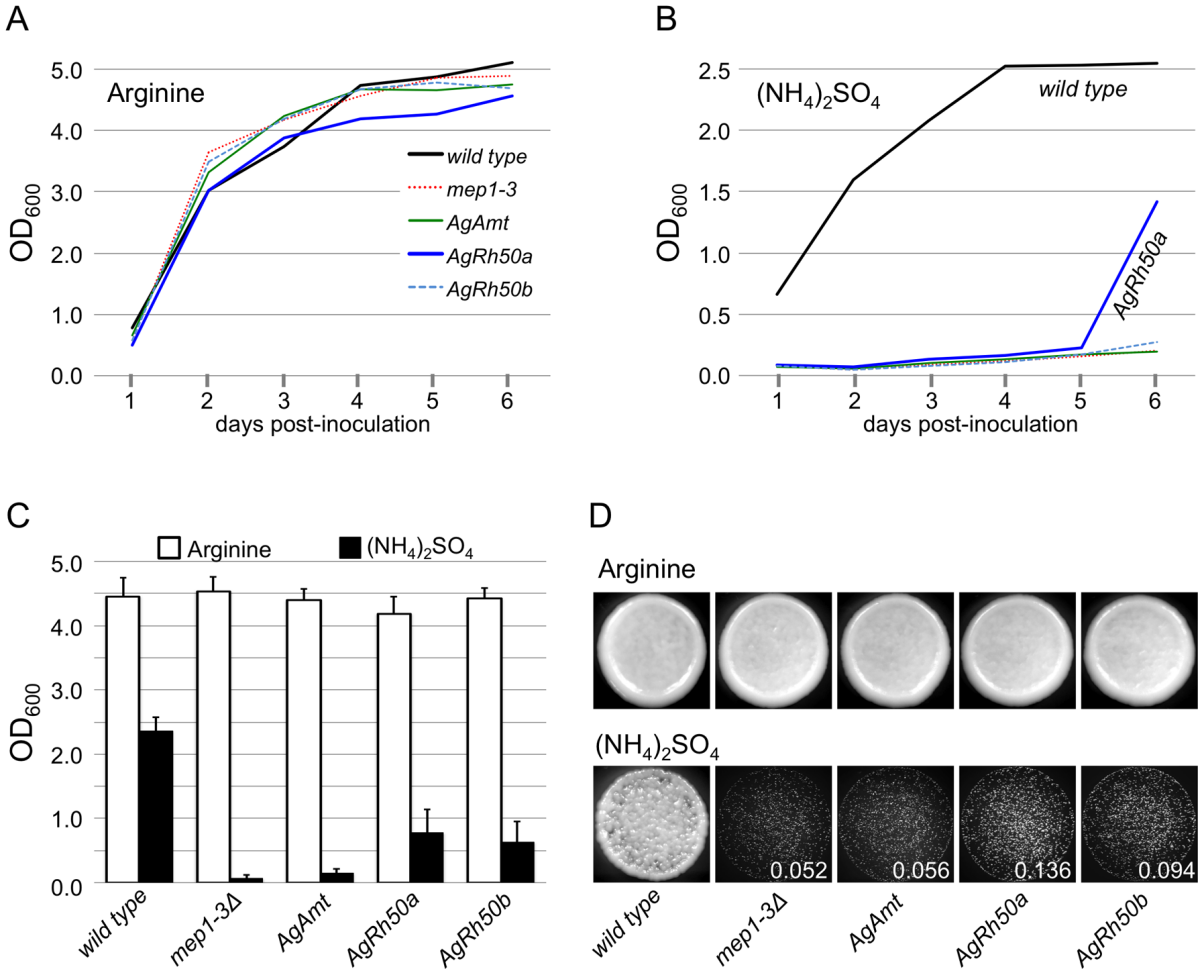
*AgRh50* Complements a Yeast Ammonium Transporter Mutant. Representative growth curve of wild type yeast and *mep1-3Δ* triple mutant transformants in minimal medium supplemented with 1 mM Arginine (**A**) or 1 mM ammonium sulfate (**B**) as the sole nitrogen source. (**C**) Histogram plot of mean optical densities for yeast transformants grown in minimal medium supplemented with 1 mM Arginine (white bards) or 1 mM ammonium sulfate (black bars). Error bars are SEM; n = 4. (**D**) Growth of yeast transformants spotted onto solid minimal medium with indicated supplements as above. Circles are 10 ul spots of ∼1000–2000 cells each, showing confluent growth on 1 mM Arginine (top panels) and reduced growth on 1 mM ammonium sulfate (bottom panels). Mean colony area (mm^2^) indicated in lower corner of bottom panes.

Given the lack of function of *AgRh50* transcripts in *Xenopus* oocytes, we attempted to complement a *S. cerevisiae* ammonium transporter triple mutant with plasmids expressing the *An. gambiae* ammonium transporters, *AgAmt*, *AgRh50a* and *AgRh50b* under the control of the galactose promoter. In these studies the *mep1-3Δ* mutant lacks all 3 endogenous ammonium transporters and accordingly grows very poorly in media where ammonium salts are the only source of free nitrogen (Figure 7). When the *An. gambiae* ammonium transporters were expressed in the mutant background, partial complementation of the *mep1-3Δ* mutant was observed for *AgRh50a* and *AgRh50b* both in liquid and solid media (Figure 7). Although not restored to wild-type levels, clear improvement in growth of the mutant strain was consistently observed after 4–6 days of growth in liquid culture (Figure 7 B,C) and after 2–4 days on solid plates (Figure 7D). In addition to improved growth based on optical density measurements of liquid cultures, colony size was similarly increased on solid media, being approximately 2–3 times larger for *AgRh50b* and *AhRh50a*, *respectively* (Figure 7D). The average optical density of *AgRh50a*-expressing strains compared with the triple mutant was significant (p = 0.08) at a reduced constraint P<0.1 in a Mann-Whitney U test (Figure 7C). We did not observed complementation of the mutant phenotype in *AgAmt* transformants.

## Discussion

We have used molecular approaches to identify *AgAmt* and *AgRh50* as ammonium transporter homologs with discrete transcripts (Figures 1–3) that are expressed in the antennae of host-seeking female *An. gambiae* mosquitoes. Interestingly, both the *AgAmt* and *AgRh50b* transcripts were highly enhanced in female antennae compared to bodies, palps or heads (devoid of antennae and palps), in multiple independent analyses (Table 1, Figure 4). Conversely, the *AgRh50a* transcript was more uniformly expressed with a lesser enhancement in heads (Table 1, Figure 4). These findings suggests partitioning of function between different isoforms of the resulting peptides, perhaps reflecting a more critical role for *AgRh50a* in ammonia clearance in the head and body and more specialized sensory roles for *AgAmt* and *AgRh50b* in the antennae. One possibility is that *AgAmt* and *AgRh50b* are expressed in the support cells surrounding ORNs and are involved in the clearance of ammonia from the aqueous lymph of antennal sensilla. This would serve to effectively reduce the noise associated with low constant exposure to environmental ammonia and would consequently allow the mosquito to maintain a high threshold of sensitivity to changes in ammonia concentration. Another possibility is that one or both ammonia transporters are localized within dendritic membranes of ORNs and directly facilitate rapid and highly effective transport of ammonia/ammonium into the cells, thereby evoking membrane potential changes that may lead to action potential firing via an unknown signaling mechanism(s). In either case, these models represent important and perhaps essential roles for this class of transporters in maintaining mosquito sensitivity to ammonia. Further studies will be required to resolve the exact functions of *AgAmt* and *AgRh50* in physiological and behavioral responses of *An. gambiae* females to ammonia.

We have also used heterologous expression and TEVC analysis in oocytes to examine the ammonium transporter functionality of *AgAmt* and the two *AgRh50* isoforms. While previous studies have found that uninjected, clamped oocytes display inward currents in response to ammonium concentrations greater than or equal to 1 mM [48], [49], we demonstrated that *AgAmt* cRNAs are sufficient to evoke dose dependent inward currents to ammonium at concentrations well below 1 mM (Figure 5A). In our studies, injection of *AgAmt* cRNAs into oocytes subsequently assayed with TEVC yielded dose dependent inward currents to ammonium chloride concentrations ranging from 200 nM to 200 µM when voltage clamped at −80 mV (Figure 5A). We interpret these dose-dependent currents as evidence of functional *AgAmt* complexes in oocytes, as currents were not observed in buffer-injected control oocytes when the same concentrations of ammonium chloride were presented (Figure 5A).

We also confirmed that *AgAmt*-injected oocytes display inward currents larger in magnitude than buffer-injected controls in response to higher concentrations of ammonium >1 mM (Figure 5B). Assuming that only these two ammonium-induced currents are present in *AgAmt*-injected oocytes, subtracting the control inward currents from the currents recorded from *AgAmt*-injected oocytes would yield the net contribution of *AgAmt* complexes to each ammonium chloride concentration tested. Pharmacological analysis aimed towards inhibiting the individual currents will be necessary to further support this conclusion.

Other functionally characterized Amts have the ability to transport methylammonium, a slightly larger derivative of ammonium, although with less efficacy and/or potency [53], [55], [56]. We also observed inward currents in *AgAmt*-injected oocytes in response to methylammonium chloride (Figure 5C). 200 µM methylammonium chloride evoked inward currents in voltage clamped *AgAmt*-injected oocytes that were approximately 55% of the magnitude of ammonium at the same concentration (Figure 5C). In addition to testing ammonium chloride and methylammonium chloride, Na^+^, K^+^, and Ca^2+^ chloride salts were tested at 200 µM to determine if chloride was eliciting the observed currents (Figure 5C). Neither *AgAmt*-injected oocytes, nor control oocytes elicited currents when perfused with Na^+^, K^+^, and Ca^2+^ chloride salts at 200 µM (Figure 5C). These findings strongly suggest that *AgAmt* is capable of transporting both ammonium and the larger derivative, methylammonium, across biological membranes, thus increasing our confidence that *AgAmt* can function as an ammonium transporter *in vivo*, although the exact mechanisms and consequences of its transport activity in *An. gambiae* tissues remains unresolved.

When oocytes were injected with either *AgRh50a* or *AgRh50b* cRNAs, the TEVC response profiles were not different from buffer-injected controls in response to varying concentrations of ammonium chloride and methylammonium chloride (data not shown). The lack of inward currents observed from *AgRh50*-injected oocytes could be the result of missing protein co-factors required for the formation of functional *AgRh*50 complexes. Alternatively, functional *AgRh50* complexes may not be electrogenic, like some mammalian Rh50 transporters, such that their activity cannot be detected within the TEVC assay [8].

In yeast growth assays, we observed at least partial complementation of a triple mutant yeast strain when expressing *AgRh50a* and a trend toward improved growth when expressing *AgRh50b* (Figure 7). These results support the conclusion that both AgRh50 proteins are able to function as ammonium transporters in yeast cells, albeit at a reduced efficiency compared to the endogenous yeast proteins. Incomplete complementation by *An. gambiae* ammonium transporters could simply reflect the inability of a single subunit to compensate for the loss of all 3 endogenous genes. In addition, we could not control for expression levels, transport/membrane localization or differences in efficiencies of ammonium transporters that may be a consequence of divergences in their primary sequences.

Taken together, these data provide compelling evidence that *AgAmt* and *AhRh50* are functional ammonium transporters. The abundance and antennal localization of both *AgAmt* and *AgRh50* suggest they may have specialized functions in sensory physiology of *An. gambiae* antennae where they mediate responses to ammonia, arguably one of the most important odor cues utilized by host seeking female mosquitoes. Given the eroding efficacy of current mosquito control methods, which rely heavily on the use of insecticides for mosquito control, the identification of novel sensory mechanisms may ultimately lead to the development of new tools for individual personal protection or surveillance that can help reduce disease transmission.

## Supporting Information

Table S1 **Ammonium transporters sampled from a broad range of species.** Peptide sequences were used in the construction of the phylogenetic tree in Figure 3.
(CSV)
